# Expression of a Y-located human proto-oncogene *TSPY* in a transgenic mouse model of prostate cancer

**DOI:** 10.1186/2045-3701-4-9

**Published:** 2014-02-17

**Authors:** Tatsuo Kido, Stephanie Schubert, Shingo Hatakeyama, Chikara Ohyama, Jörg Schmidtke, Yun-Fai Chris Lau

**Affiliations:** 1 Laboratory of Cell and Developmental Genetics, Department of Medicine, VA Medical Center & Institute for Human Genetics, University of California, 4150 Clement Street, San Francisco, CA, USA; 2 Institute of Human Genetics, Hannover Medical School, D-30625 Hannover, Germany; 3 Department of Urology, Hirosaki University Graduate School of Medicine, Hirosaki 036-8562, Japan

**Keywords:** Prostate cancer, Y-chromosome, Proto-oncogene, Transgenic mouse models, *TSPY*

## Abstract

**Background:**

The human *TSPY* is the putative gene for the gonadoblastoma locus on the Y chromosome (GBY). Various molecular, pathological and transgenic mouse studies suggest that *TSPY* is a Y-located proto-oncogene contributing to the initiation/progression in human cancers, including germ cell tumors and various somatic cancers, such as prostate and liver cancer, and melanoma. The TgTSPY9 transgenic mouse line harbors a 8.2-kb human *TSPY* structural gene, which is tandemly integrated in the mouse Y chromosome, and expressed in a similar pattern as that of the endogenous gene in the human genome. This mouse model of human *TSPY* gene offers an opportunity to examine its behavior and potential contribution in various mouse models of human diseases, such as human cancers. We had investigated the expression of such *TSPY*-transgene in the LADY mouse model of prostate cancer, harboring a SV40 T antigen gene directed by a rat probasin promoter; and compared the expression pattern with those of endogenous *TSPY* gene and biomarkers in human prostate cancer specimens.

**Results:**

By introducing the Y-located *TSPY*-transgene to the LADY mice, we had examined the expression pattern of the human *TSPY* during prostatic oncogenesis in this mouse model of prostate cancer. Our results showed that the *TSPY*-transgene was activated in selected areas of the hypercellular stroma but not in the intraepithelial cells/neoplasia in the prostates of TgTSPY9/LADY mice. Using a specific biomarker, FOXA1, for epithelial cells, we demonstrated that TSPY-positive cells proliferated exclusively in the cancerous stroma in the LADY model at late stages of tumorigenesis. In contrast, in the human situation, TSPY was predominantly co-expressed with FOXA1 in the epithelial cells of PIN lesions and FOXA1 and another cancer biomarker, AMACR, in the adenocarcinoma cells in clinical prostate cancer samples of various degrees of malignancy.

**Conclusions:**

Our data show that human *TSPY* could be abnormally activated during prostatic oncogenesis, and could possibly contribute to the heterogeneity of prostate cancer. The differential expression patterns of the human *TSPY* between the LADY mouse model and clinical prostate cancer suggest potential limitations of current mouse models for studies of either *TSPY* behavior in diseased conditions or prostate cancer development.

## Background

Prostate cancer is one of the most frequently diagnosed cancers among men. More than 200,000 new cases are diagnosed each year, and more than 30,000 people die from prostate cancer in United States in 2011 (report of National Cancer Institute, United States) [1]. The etiology of prostate cancer is currently uncertain, and might follow a gradual transformation of normal prostate epithelium to prostatic intraepithelial neoplasia (PIN), and to locally invasive carcinoma and metastatic disease [2,3]. Prostate cancer initiation, development and progression are complex processes, involving multiple environmental and genetic risk factors and co-carcinogenic processes between epithelia and stroma of the prostate at different stages [4-8]. Since prostate cancer is a man-specific cancer, the contribution of genes on the male-only Y chromosome have been suspected, but remains controversial due to contradictory observations on the gain and loss of this chromosome in samples of numerous tumor types [9-13]. The complete sequencing of the human genome [14], particularly the Y chromosome [15], has provided significant information on the genetic content of the human Y chromosome; thereby providing an opportunity to evaluate independently the various genes on this male-specific portion of the human genome.

Among the genes on the human Y chromosome, the testis-specific protein Y-encoded (*TSPY*) gene represents the most likely gene potentially contributing to the complex etiology of prostate cancer [16]. *TSPY* is a repeat gene, mapped to the critical region within the gonadoblastoma locus on the human Y-chromosome (GBY), which serves a normal function in the testis, but could predispose the dysgenetic gonads of patients of disorders of sexual development (DSD) to develop gonadoblastoma [17-21]. Indeed, *TSPY* is expressed in normal germ cells, and abundantly and frequently expressed in various types of germ cell tumors such as gonadoblastoma, carcinoma in situ/intratubular germ cell neoplasia unclassified (CIS/ITGCNU), and seminoma [20-24]. Importantly, it is also expressed in various types of somatic cancer, including prostate cancer, hepatocellular carcinoma, and melanoma [25-28]. Hence, TSPY is considered as one of cancer/testis antigens as a potential candidate in vaccine strategy for immunotherapy in cancer patients [26]. TSPY interacts with the type B cyclins and enhances the cyclin-B/CDK1 activity [29]. It exhibits oncogenic properties when transfected to NIH3T3 cells [30]. Further, TSPY also interacts with various regulatory proteins, such as translation elongation factor EEF1A [31], and promotes cell growth and proliferation associated with cancer development. Hence, understanding its expression patterns could aid the development of diagnostic and therapeutic strategies in medical management of various cancers affected by this Y-located proto-oncogene.

Various mouse models of prostate cancer have been generated to investigate the susceptibility, initiation, progression and pharmacology of the disease [32-34]. The LADY (also termed as LPB-Tag) transgenic mouse line is one of these mouse models. It harbors a SV40 large T antigen (Tag) oncogene directed by a 12-kb rat probasin promoter, which is expressed at high levels in the prostates of the host animals [35]. The LADY mice develop epithelium dysplasia, carcinoma-in-situ and adenocarcinoma in their prostates, similar to human prostate cancer [35]. The LADY model consists of several distinct transgenic mouse strains with different transgene integration sites. The 12T-7f LADY is a fast disease-developing strain, and develops complex mouse prostatic intraepithelial neoplasia (mPIN) with hypercellular stroma by 20 weeks of age [36,37].

Transgenic modeling of *TSPY* in oncogenesis has been difficult. Although it is evolutionarily conserved on the Y chromosome among various mammalian species including apes and bovine [38,39], the mouse *Tspy* is a non-functional single-copy gene [40,41]. Transgenic mice harboring a human *TSPY* gene on their autosome(s) are difficult to maintain as transgenic mouse lines, potentially caused by its inappropriate expression and disruptive effects on the reproductive system or embryonic development of the host animals [42]. Recently, a transgenic mouse line, designated as TgTSPY9 harboring the human *TSPY* gene on the mouse Y-chromosome, has been established [43]. The TgTSPY9 mice harbor an 8.2-kb genomic fragment derived from the human Y chromosome, containing the 2.8-kb TSPY transcriptional unit flanked by 2.95-kb promoter and 2.45-kb 3′ sequences. The 8.2-kb *TSPY*-transgene is integrated tandemly about 50 copies on the Y chromosome of the host genome. The organization of the *TSPY*-transgene on the mouse Y chromosome resembles that of the endogenous *TSPY* gene in the human genome, which is tandemly repeated ~ 20–60 times on the Y chromosome. Significantly the *TSPY*-transgene is specifically expressed in pre-spermatogonial cells in embryonic testis and spermatogonial and prophase I germ cells in adult testis, while little or no expression is detected in other somatic tissues, including non-cancerous prostate [42,43], similar to the expression pattern observed for the endogenous *TSPY* gene in humans [42,44]. Hence, by integrating onto the host Y chromosome, the *TSPY*-transgene is properly regulated as the endogenous gene in the human genome, thereby establishing the TgTSPY9 transgenic line as a suitable mouse model for the human *TSPY* gene in studies, designed to determine its functions in various biological and experimental systems.

In the present study, we have explored the potential involvement of *TSPY* in the oncogenic processes of the LADY transgenic mouse model of prostate cancer. Our results suggest that *TSPY* could be ectopically activated and expressed in the oncogenic process(es) in this model of prostate cancer. *TSPY* expression parallels those of other biomarkers, but its pattern could be different from those observed in clinical specimens of prostate cancer patients, suggesting the potential heterogeneity of oncogenic processes in both the LADY model and human prostate cancer.

## Results

### The human *TSPY*-transgene was expressed in the hypercellular stroma of TgTSPY9/LADY mouse prostate

The LADY and TgTSPY9 mice were originally maintained on CD1 and NMRI background respectively [35,43]. Since tumor development in mouse model is frequently affected by genetic background [45-47], we first analyzed the prostatic tumor development of LADY mice on CD1/NMRI F1 hybrid genetic background. Prostatic enlargement was observed at 12 weeks of age and the tumor size reached to ~ 2 cm in diameter at 19 weeks, an experimental endpoint in our animal protocol. Both mouse prostatic intraepithelial neoplasia (mPIN) and hypercellular stroma and were observed in LADY mice in CD1 and CD1/NMRI background while glands were surrounded by normal appearing fibromuscular stroma in non-transgenic CD1 and CD1/NMRI F1 mouse prostate. These preliminary observations suggest that the prostatic oncogenic processes in the CD1/NMRI background were similar as those initially described in CD1 background for the LADY model of prostate cancer [35,36].

**Figure 1:**
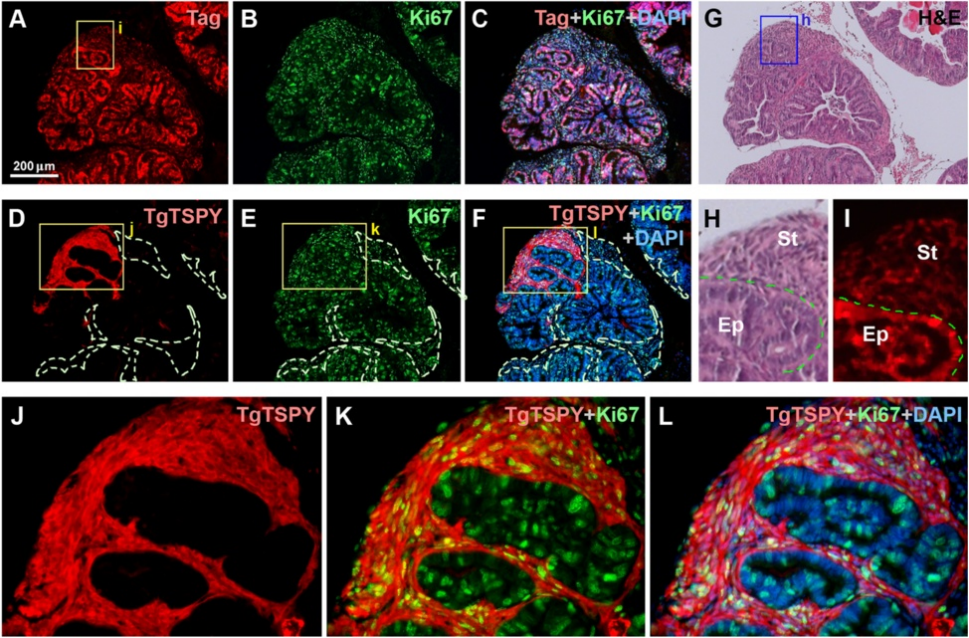
**Expression of *TSPY *transgene in the prostates of TgTSPY9/LADY mice at 12 weeks of age. A-C**, Double immunofluorescence staining of T-antigen (red) and Ki67 (green) in the prostate of TgTSPY9/LADY mouse at 12 weeks of age. DNA was visualized by DAPI staining (blue). **C** represents the merged image of T-antigen (red), Ki67 (green) and DNA (blue). **D-F**, Double immunofluorescence staining of the TSPY-transgene product (TgTSPY, red) and Ki67 (green) on the adjacent section of **A-C**. **F** represents the merged image of TgTSPY (red), Ki67 (green) and DNA (blue). TgTSPY-negative hypercellular stroma areas were circled by dot lines. **G-H**, H&E staining of an adjacent section of **A-C**. **H** shows the highly magnified image of the boxed area in **G**. Epithelial cells (Ep) and hypercellular stroma (St) were labeled. **I** is a highly magnified view of the boxed area in A, staining of T-antigen. T-antigen was expressed in both epithelial cells and stromal cells. **J-L**, Highly magnified views of TgTSPY (red) and Ki67 (green) for the boxed area in **D-F** respectively. **K** represents the merged view of TgTSPY (red) and Ki67 (green), and **L** represents the merged image of **K** plus DNA staining (blue). Scale bar = 200 μm in **A-G**. Significant expression of TgTSPY was frequently detected in hypercellular stroma, developed in the prostate of TgTSPY9/LADY mice at this age.

To explore the potential role of the *TSPY*-transgene in the LADY model of prostate cancer, we crossed TgTSPY9*/*NMRI male mice and LADY/CD1 heterozygous female mice, and analyzed the F1 generation male offspring at various ages. At 12 weeks of age, small but pronounced hypercellular stroma was frequently observed in addition to the progressive expansion of atypical epithelium in the prostates of TgTSPY9/LADY mice (Figure 1G and H). Immunofluorescence analysis demonstrated that large T antigen and Ki67, a cell proliferative marker, were expressed in the majority of cells in both epithelial and stromal cells, as observed in LADY/NMRI mice (Figure 1A-C and I). The *TSPY*-transgene product was locally expressed in clusters of cells in the hypercellular stroma regions (Figure 1D-F and J-L) but not the adjacent tissues.

**Figure 2:**
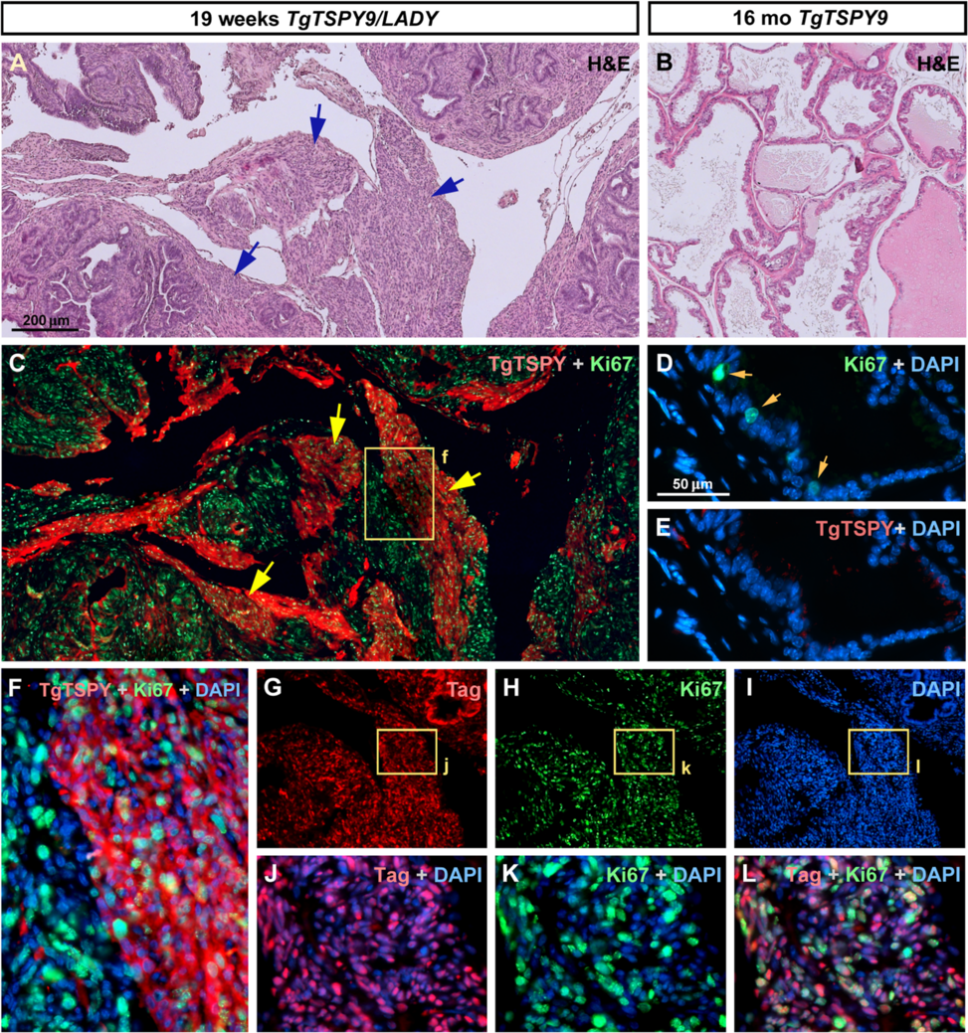
**Expression of *TSPY*-transgene in the prostates of TgTSPY9/LADY mice at 19 weeks of age. A**, Representative H&E staining image of the sections of prostate from TgTSPY9/LADY mouse at age 19 weeks. Hypercellular stroma was further enlarged comparing with 12 weeks of age (arrows). **B**, H&E stained image of the sections of prostate from TgTSPY9 mouse at 16 months of age. **C**, Double immunofluorescence staining of the *TSPY*-transgene product (TgTSPY, red) and Ki67 (green) in the adjacent section of **A**. TgTSPY was significantly expressed in large part of hypercellular stroma. **D-E**, Double immunofluorescence staining of TgTSPY (red) and Ki67 (green) in the prostate from TgTSPY9 mouse at 16 month of age. **D** represents a merged image of Ki67 (green) and DNA (blue), and **E** represents a merged image of TgTSPY (red) and DNA (blue). **F** represents a highly magnified view of the boxed area in **C**, showing signals of TgTSPY (red), Ki67 (green) and DNA (blue). **G-I**, Double immunofluorescence staining of T-antigen (Tag) and Ki67 in the prostate from TgTSPY9/LADY mouse at age 19 weeks (adjacent section of **C**). DNA was visualized by DAPI staining (blue). **J-L**, Highly magnified views of boxed areas in **G-I**. **J** and **K** represents the merged images of Tag (red) and DNA (blue), or Ki67 (green) and DNA (blue), respectively. **L** represents the merged view of Tag (red), Ki67 (green) and DNA (blue). Scale bar = 200 μm in **A-C**, **G-I**, and 50 μm in **D-E**. Tag and Ki67 expressed closely in same cells of the prostate of TgTSPY9/LADY mouse at age 19 weeks.

At 19 weeks of age, both Tag and Ki67 were expressed in the majority of prostatic cells and the enlarged hypercellular stroma (Figure 2A, G-L), while the clusters of TgTSPY-positive cells expanded further with the hypercellular stroma (Figure 2C and F), but not any mPIN lesions. In contrast, no hyperplasia was observed and no obvious TgTSPY-positive cell was detected by immunofluorescence in the prostate of a single-transgenic TgTSPY9/NMRI mouse even at 16 month of age (Figure 2D-E). Such expression pattern of the *TSPY*-transgene in LADY mice was different from that of the endogenous human *TSPY* in clinical prostate cancer samples [27,28]. These observations suggested that the behavior of *TSPY*-transgene in the TgTSPY9/LADY mice is likely to be different from that of human *TSPY* in prostate cancer.

### TSPY and FOXA1 are co-expressed in the same tumor cells in human prostate cancer, but not in those of the LADY model of prostate cancer

**Figure 3:**
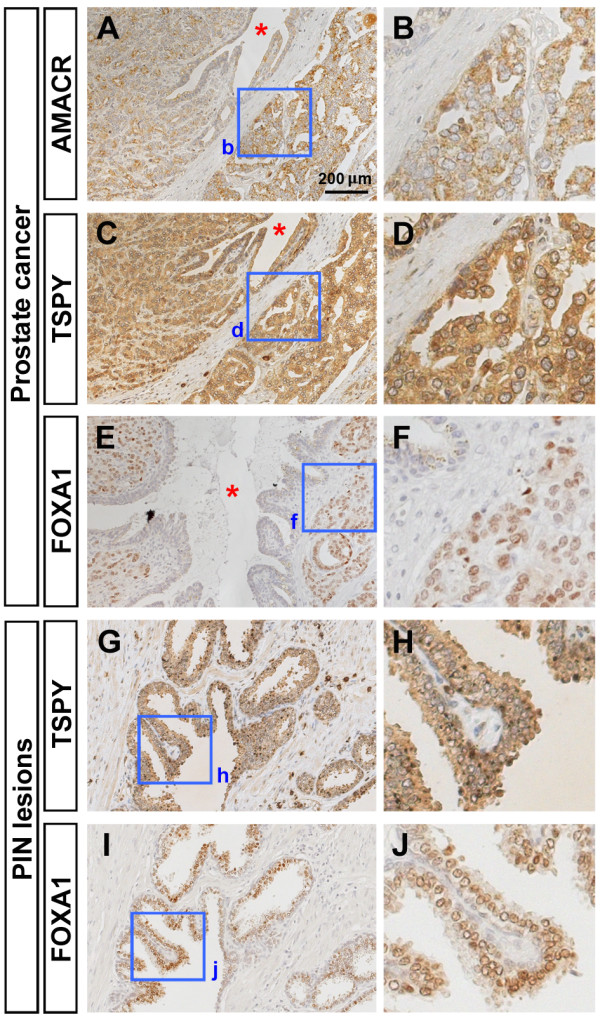
**Immunohistochemical analysis of TSPY, FOXA1 and AMACR expression in human prostatic cancer specimens. A-B**, Immunostaining of AMACR (brown) in prostatic adenocarcinoma. Nuclei were counterstained by hematoxylin (blue). **C-D**, Immunostaining of TSPY (brown) in prostatic adenocarcinoma (adjacent section of **A**). Significant TSPY staining was observed in cancer cells, but faint or negative staining in the surrounding stroma. **E-F**, Immunostaining of FOXA1 (brown) in prostatic adenocarcinoma (section close to **A** in the same specimen). Strong signal of FOXA1 expression was observed in nuclei of adenocarcinoma cells. Asterisks indicate the common duct among **A**, **C** and **E**. **G-H**, Immunostaining of TSPY (brown) in PIN lesions. **I-J**, Immunostaining of FOXA1 (brown) in PIN lesions (adjacent section of **G**). Both *TSPY* and FOXA1 were expressed in adenocarcinoma cells and epithelial cells in PIN lesions. **B**, **D**, **F**, **H** and **J** represent the highly magnified views of the boxed area in **A**, **C**, **E**, **G** and **I**, respectively. Scale bar = 200 μm in **A**, **C**, **E**, **G** and **I**.

The preferential location of the *TSPY*-transgene product in the hyperplastic stromal cells was somewhat different from our previous observations in clinical and latent prostate cancer specimens, in which the TSPY protein were mostly localized in the epithelial tumor cells [27,28]. To confirm such differential cytological locations of TSPY in both clinical samples and LADY model of prostate cancer, we have examined the TSPY expression with reference to the forkhead class DNA-binding protein A1 (FOXA1) biomarker, which is preferentially expressed in prostatic epithelial cells and play crucial roles in prostate cancer initiation and development in both human specimens and mouse models [48-50]. In human clinical prostate cancer specimens, TSPY was predominantly detected in the epithelial tumor cells, which were also positive for the tumor biomarker, alpha-methylacyl-CoA racemase (AMACR) [28]. TSPY was strongly expressed in adenocarcinoma cells positive for AMACR (Figure 3A-D). In the adjacent tissue sections, nuclei of those TSPY-positive adenocarcinoma cells were also positively stained by anti-FOXA1 (Figure 3E-F). Further, both TSPY and FOXA1 proteins were co-expressed and co-localized in the same hyperplastic epithelial cells of PIN lesions (Figure 3G-J), suggesting that TSPY could exacerbate the oncogenic properties of FOXA1 and other cancer-promoting biomarkers in prostatic epithelial and tumor cells during cancer development and progression.

**Figure 4:**
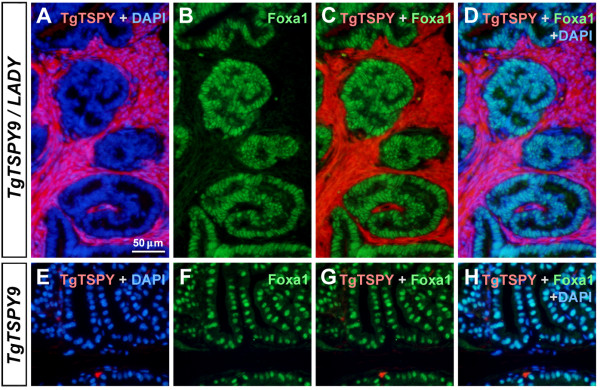
**Expression of *TSPY*-transgene and Foxa1 in the prostates of TgTSPY9/LADY mice at 14 weeks of age. A-D**, Double immunofluorescence staining of the *TSPY*-transgene product (Tg*TSPY*, red) and Foxa1 (green) in the prostate of TgTSPY9/LADY mouse at 14 weeks of age. DNA was visualized by DAPI staining (blue). **C** represents the merged view of TgTSPY (red) and Foxa1 (green), and D represents the merged view including DNA (blue). Note that the expression of TgTSPY did not overlap with that of Foxa1. **E-H**, Double immunofluorescence staining of TgTSPY (red) and Foxa1 (green) in the prostate of singly transgenic TgTSPY9 mouse at 14 weeks of age. **G** represents the merged view of TgTSPY (red) and Foxa1 (green), and **H** represents the merged view including DNA (blue). Scale bar = 50 μm in **A-H**.

To further investigate the distribution of *TSPY*-transgene expression in TgTSPY9/LADY mice, we performed double immunofluorescence analyses of *TSPY* and *Foxa1* endogenous gene products in the prostates of TgTSPY9*/*LADY bi-transgenic and TgTSPY9/CD1 single-transgenic mice at 14 weeks and 19 weeks of age. At both ages, mouse Foxa1 was predominantly expressed in the nuclei of atypical epithelium in TgTSPY9*/*LADY mice and those of normal epithelial cells in the single-transgenic TgTSPY9/CD1 mice (representative images at 14 weeks of age in Figure 4B and F respectively). In contrast, the *TSPY*-transgene was expressed in hyperplastic stromal cells in TgTSPY9*/*LADY mice, and no overlap in the respective immunofluorescence staining between the human *TSPY* transgene and the mouse Foxa1 endogenous gene products was observed in the merged images (Figure 4C-D). In addition, no obvious TgTSPY-positive cells were detected in the TgTSPY9/CD1 mouse prostate at 14 weeks of age (Figure 4E-H). These observations suggest that, TSPY is differentially expressed in LADY transgenic mouse model of prostate cancer and clinical human prostate cancer.

## Discussion

**Figure 5:**
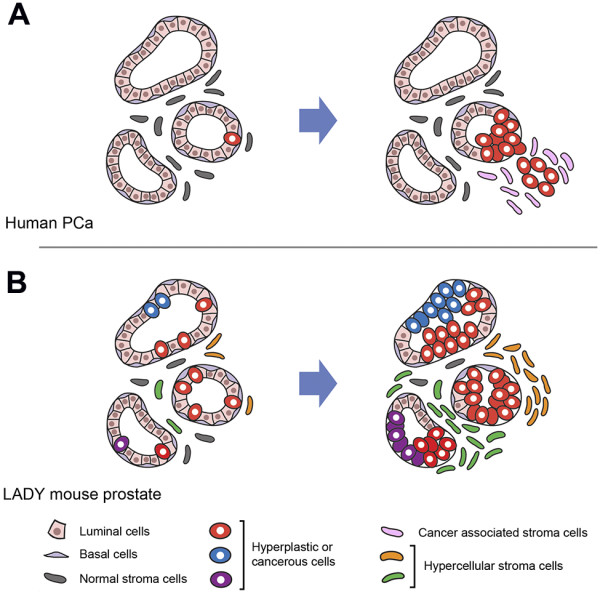
**Schematic diagram illustrating potential patterns of human prostate cancer development and the neoplastic prostate growth in LADY mouse. A**, Human prostate cancer originates from prostate epithelial cells; either basal cells or luminal cells [51-53]. Although primary prostate cancer is regarded as multifocal, advanced prostate cancer is postulated to be monoclonal [3]. Cancer associated stromal cells proliferate in the oncogenic processes [60]. **B**, In the LADY-mouse prostate, ubiquitously expressed T-antigen oncogene enables various types of cells, including stromal cells, to be hyper-proliferative and oncogenic in multiple regions. As the results, cancer could originate from multiple types of progenitor cells.

The role of the Y chromosome in prostatic oncogenesis has been debated numerous occasions, in which the gain or loss of this male-only chromosome has been reported [9-13]. The identification of *TSPY* as the gene for the gonadoblastoma locus on the Y chromosome has raised the possibility that this repeated proto-oncogene could participate in the oncogenic processes of human cancers, in which *TSPY* is inappropriately expressed. The present study represents an assessment of the TgTSPY9 as a model for evaluation of the response of the Y-located *TSPY*-transgene to experimental oncogenesis. Human prostate cancer has been postulated to originate from prostate epithelial cells (either basal cells or luminal cells) [51-53] (Figure 5). *TSPY* is normally expressed in the spermatogonial cells and prophase I of the adult testis, but not in somatic cells. However, under oncogenic conditions, *TSPY* is activated in epithelial cells in the morphologically normal glands adjacent to cancer area, as well as in adenocarcinoma cells in prostate cancer [27,28]. Previously, we demonstrated that TSPY could exert pro-growth properties in enhancing protein synthesis, accelerating cell cycle progression, stimulating cyclin B-CDK1 kinase activities, abbreviating G_2_/M transition, which potentially could abolish G_2_/M checkpoints and increasing chromosome nondisjunction and genomic instability [29,30]. Hence, *TSPY* activation in the premalignant epithelial cells, and tumorigenic cells could collectively promote prostatic carcinogenesis. However, no significant difference was observed in the size of prostates and tumors between LADY and TgTSPY9/LADY mice in the present study (data not shown). Currently, the exact reason is uncertain. Activation of TSPY expression in the hyperplasia stroma could have an effect(s) on the evolution of the tumorigenic processes not associated with tumor size. Significantly, some studies suggest that the SV40-based transgenic models of oncogenesis have limitations, since the SV40 viral oncoprotein is not a likely etiologic agent for prostate cancer in humans and it has relatively intensive cellular effects [54], such that the mice, e.g. LADY mice, rapidly developed massive prostatic hyperplasia within a few months. Human primary prostate cancer, on the other hand, is slow growing in most cases [55,56]. We surmise that the effects of TSPY might not be readily noticeable in fast-growing tumor developed in the LADY mouse background. Mouse models that slowly develop prostatic tumor should be helpful in elucidating the role of TSPY in cancer development *in vivo*.

The activation of the Y-located *TSPY*-transgene in the prostate of the LADY model seems to validate the postulation that human *TSPY* gene on the Y chromosome could be dysregulated and activated under prostatic oncogenic conditions. The present observation that the *TSPY*-transgene is expressed in hypercellular stroma but not in mPIN lesions in the prostate of TgTSPY9*/*LADY mice is somewhat unexpected from the initial postulation. At present, the exact mechanism(s) contributing to such differential *TSPY* expression patterns between human prostate cancer and LADY tumor model is uncertain. It has been reported that the gene expression pattern is largely different between mouse prostate and human prostate [57]. In addition, prostatic epithelial cells and stromal cells express specific sets of genes different from each other during oncogenesis [57,58]. We postulate that a combination of species-specific and cell type-specific gene regulation mechanisms could be involved in the differential expression of *TSPY* between human clinical prostate cancer and TgTSPY9/LADY mouse prostatic tumor.

Human prostate cancer is postulated to originate from epithelial cells (either basal cells or luminal cells) of the prostate [51-53], however, numerous studies have clearly demonstrated that the stromal cells also play key roles to the overall carcinogenesis [6-8,59,60] (Figure 5). Hence interplay between epithelial and stromal cells could be important in human prostatic oncogenesis. In the LADY model of prostate cancer, the SV40 large T antigen is ubiquitously expressed in the epithelial and stromal cells of the prostate and might possibly exert its oncogenic actions in both cell types. Our results suggest the *TSPY*-transgene could be more susceptible for being activated from its tight Y chromosome regulation in stromal cells than epithelial cells in the prostates of the LADY mice. Since all TSPY-positive cells are stromal cells at later stages of the prostatic tumors, these TSPY-positive stromal cells could have a clonal origin. The fact that only clusters of stromal cells are positive for TSPY at all stages suggests heterogeneity in the tumorigenic processes in the LADY model of prostate cancer. The differential expression of the Y-located human TSPY gene between the LADY mouse model and clinical prostate cancer highlights a possible limitation(s) of current modeling of human prostate cancer in mice.

## Materials and methods

### Animals

Mice for the 12T-7f LPB-Tag (LADY) model of prostate cancer were obtained from the NCI-Mutant Mouse Repository (MD, USA), and were initially constructed and characterized by Matusik and colleagues [35]. TgTSPY9 transgenic mouse line, harboring multiple copies of 8.2 kb genomic fragment of human TSPY gene on its Y-chromosome, was originally constructed by Drs. Stephanie Schubert and Jorge Schmidtke [43]. The genomic background of 12T-7f LADY mice and TgTSPY9 mice were CD-1 and NMRI respectively. Tissues were dissected from 12T-7f LADY × TgTSPY9 heterozygous F1 mice, and analyzed by immunohistochemical methods as described below.

All animals were maintained as breeding colonies at the Animal Care Facility of San Francisco VA Medical Center. The Institutional Animal Care and Use Committee of the VA Medical Center approved all experimental procedures in accordance with the NIH *Guide for Care and Use of Laboratory Animals*.

### Human prostate specimens

The clinical prostate cancer specimens were derived from de-identified archival pathological preparations of the Department of Urology, Hirosaki University, Japan, and analyzed as previously described [28]. The analysis of these specimens was performed under approved protocols from the respective Institutional Committees on Human Research, VA Medical Center, San Francisco and Hirosaki University, Aomori, Japan.

### Immunofluorescence and immunohistochemical analysis

The tissues were fixed in 4% paraformaldehyde-PBS solution and embedded in paraffin by standard protocol. Immunohistological and immunofluorescence analyses of tissue sections were performed as described previously [61], using anti-SV40 T-antigen mouse antibody (1:80, Santa Cruz Biotechnology, Inc., Santa Cruz, CA, USA), anti-TSPY rabbit serum (1:400), anti-TSPY mouse monoclonal antibody (1:3000, clone-7), anti-Ki67 rabbit IgG (1:100, Neomarkers/Thermo scientific, Kalamazoo, MI, USA), anti-AMACR rabbit antibody (1:50, Abcam Inc., Cambridge, MA, USA), anti-FOXA1 rabbit antibody (1:200, Abcam Inc). The immunoreactive sites were visualized with Super Picture Polymer Detection kit (ZYMED/Invitrogen, Carlsbad, CA, USA) or VECTASTAIN Elite ABC kit (Vector laboratories, Burlingame, CA, USA) using 3, 3′-Diaminobenzidine (DAB). For immunofluorescence, the immunoreactive signals were detected by Alexa Fluor 586 (red)-conjugated anti-mouse IgG and Alexa Fluor 488 (green)-conjugated anti-rabbit IgG (Molecular Probes/Invitrogen, Carlsbad, CA, USA) as secondary antibodies. DNA was visualized by staining with 4′, 6-Diamidine,-2′-phenylindole dihydrochloride (DAPI, Roche Applied Science, Indianapolis, IN, USA). Fluorescence was examined with an Axiophoto fluorescence microscope (Carl Zeiss MicroImaging, Thronwood, NY, USA) and recorded with a LEI-750 digital imaging system (Leica Microsystmes, Bannockburn, IL, USA).

## Competing interests

The authors declare that they have no competing interests.

## Authors’ contributions

TK and YFCL conceived the concept and designed the experiments. TK performed the experiments. SS and SM constructed the TgTSPY9 transgenic mouse line and SH and CO provided the human prostate cancer specimens. TK and YFCL co-wrote the manuscript. All authors read and approved the final manuscript.
